# Disrupting Hepatocyte *Cyp51* from Cholesterol Synthesis Leads to Progressive Liver Injury in the Developing Mouse and Decreases RORC Signalling

**DOI:** 10.1038/srep40775

**Published:** 2017-01-18

**Authors:** Žiga Urlep, Gregor Lorbek, Martina Perše, Jera Jeruc, Peter Juvan, Madlen Matz-Soja, Rolf Gebhardt, Ingemar Björkhem, Jason A. Hall, Richard Bonneau, Dan R. Littman, Damjana Rozman

**Affiliations:** 1 Centre for Functional Genomics and Bio-Chips, Institute of Biochemistry, Faculty of Medicine, University of Ljubljana, Ljubljana, Slovenia; 2 Medical Experimental Centre, Institute of Pathology, Faculty of Medicine, University of Ljubljana, Ljubljana, Slovenia; 3 Institute of Pathology, Faculty of Medicine, University of Ljubljana, Ljubljana, Slovenia; 4 Institute of Biochemistry, Faculty of Medicine, University of Leipzig, Leipzig, Germany; 5 Department of Laboratory Medicine, Division of Clinical Chemistry, Karolinska Institute, Karolinska University Hospital, Huddinge, Sweden; 6 The Kimmel Center for Biology and Medicine of the Skirball Institute, New York University School of Medicine, New York, New York 10016, USA; 7 New York University & Simons Foundation for Data Analysis, New York, NY 10010, USA; 8 Howard Hughes Medical Institute, New York University School of Medicine, New York, New York 10016, USA

## Abstract

Development of mice with hepatocyte knockout of lanosterol 14α-demethylase (H^*Cyp51*−/−^) from cholesterol synthesis is characterized by the progressive onset of liver injury with ductular reaction and fibrosis. These changes begin during puberty and are generally more aggravated in the knockout females. However, a subgroup of (pre)pubertal knockout mice (runts) exhibits a pronounced male prevalent liver dysfunction characterized by downregulated amino acid metabolism and elevated *Casp12.* RORC transcriptional activity is diminished in livers of all runt mice, in correlation with the depletion of potential RORC ligands subsequent to CYP51 disruption. Further evidence for this comes from the global analysis that identified a crucial overlap between hepatic *Cyp51*^−/−^ and *Rorc*^−/−^ expression profiles. Additionally, the reduction in RORA and RORC transcriptional activity was greater in adult H^*Cyp51*−/−^ females than males, which correlates well with their downregulated amino and fatty acid metabolism. Overall, we identify a global and sex-dependent transcriptional de-regulation due to the block in cholesterol synthesis during development of the *Cyp51* knockout mice and provide *in vivo* evidence that sterol intermediates downstream of lanosterol may regulate the hepatic RORC activity.

The liver is to date the best example of a non-reproductive tissue that shows major differences in gene expression between males and females. The majority of liver-related sex differences emerge during pubertal development^1^ with adult mice exhibiting well over 1,000 genes differentially expressed (DE) between females and males^2^.

The development of liver begins at embryonic (E) day 8.5–9, with hepatocyte differentiation^3^ and cholesterol production^4^ starting around day E13.5. At birth, 55–60% of cholesterol in the liver is produced endogenously, while in adults hepatic cholesterol production provides 10% and 40% of total cholesterol in humans and mice, respectively^5^. Consequently, mouse liver knockouts of cholesterol synthesis genes survive embryonic development in contrast to the lethal phenotypes of the full knockout mice^6^. Blocking earlier steps of the pathway produces a stronger phenotype in both cases. The hepatocyte *Hmgcr*^−/−^ mice survive for up to 5 weeks^7^, while mice lacking the rate limiting step of the post-lanosterol pathway, the hepatocyte *Cyp51*^−/−^ mice, survive for over 19 weeks although with marked changes in liver structure and function^8^. Cholesterol intermediates after lanosterol have long been thought of as dedicated exclusively to cholesterol in contrast to earlier metabolites^9^. However, meiosis-activating sterols (MAS) have been identified for their importance in gametogenesis^10^ and intermediates further downstream most recently as potential ligands for RORC isoform 2 (RORγt) in Th17 cells^11^,^12^. RORC is an orphan nuclear receptor expressed in several tissues, including the liver^13^. The two isoforms have distinct functions and distribution, but with the same ligand- and DNA-binding domains^14^. RORγt is the primary isoform in thymocytes and is required for normal lymph node development^15^, whereas the more common isoform 1 (RORγ1 or RORC) modulates the circadian clock, as well as glucose and fat metabolism and in the liver peaks at circadian time ZT16–20^16^,^17^,^18^.

Herein we examine the development of the hepatocyte *Cyp51*^−/−^ mice and show that normal liver function becomes impaired by the end of puberty, with earlier presentation and enhanced liver damage in females. A subgroup of male prevalent knockout mice exhibits augmented liver dysfunction combined with abnormal hepatic sterol profiles. Lastly, our work highlights the need for unperturbed cholesterol synthesis for normal RORC signalling *in vivo.*

## Results

### Characterization of *Cyp51* ^−/−^ mice during postnatal development

Hepatocyte *Cyp51* knockout mice (H^*Cyp51*−/−^) on the wild type (*Cyp51*^+/+^)^8^ or heterozygous (*Cyp51*^+/−^) background were born in accordance with the Mendelian ratio (Supplementary Table 1) and were euthanized at 0, 3, 6, 9 and 19 weeks of age (Supplementary Fig. 1).

**Figure 1:**
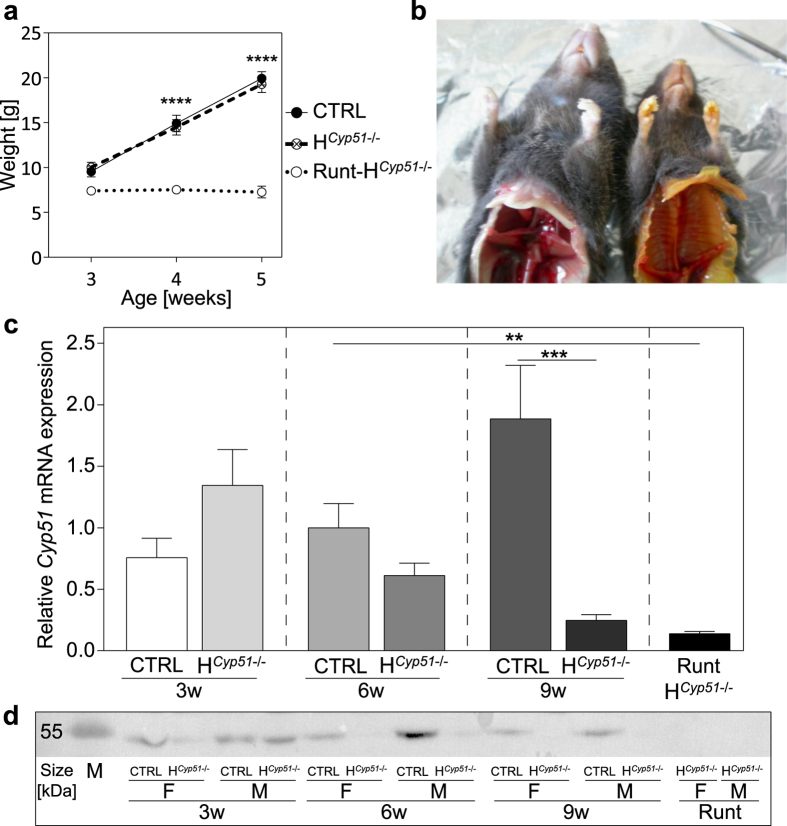
Hepatocyte deletion of *Cyp51* causes (pre)pubertal detrimental defects. (**a**) Growth curves of control (CTRL; n ≥ 6), H^*Cyp51*−/−^ (n ≥ 6) and runt mice (5–7 weeks; n ≥ 3) on a wild type or heterozygous *Cyp51* background. Error bars represent SEM. (**b**) A representative picture of a male H^*Cyp51*−/−^ and a male runt mouse after autopsy. (**c**) A comparison of relative *Cyp51* expression between control, H^*Cyp51*−/−^ and runt mice on a wild type or heterozygous *Cyp51* background at different ages (n ≥ 8). Columns depict means and error bars represent SEM. (**d**) CYP51 protein expression as measured by western blot analysis. Mice were grouped based on age, sex and genotype (regardless of the *Cyp51* background). **p < 0.01; ***p < 0.001; ****p < 0.0001.

**Figure 2:**
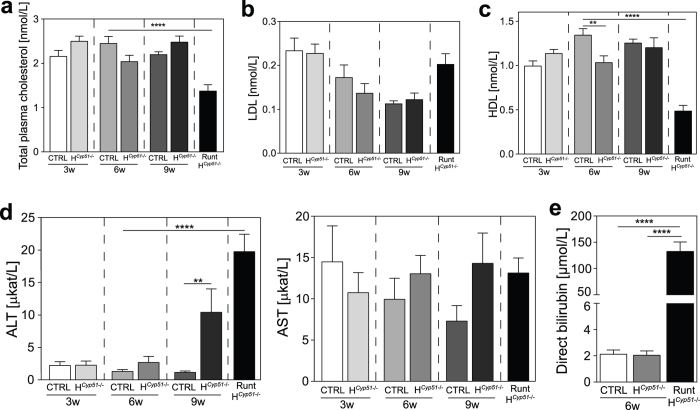
Plasma analyses reveal altered cholesterol homeostasis in runts. (**a**) Total plasma cholesterol, (**b**) LDL cholesterol, (**c**) HDL cholesterol and (**d**) ALT and AST concentrations in control, H^*Cyp51*−/−^and runt mice on a wild type or heterozygous *Cyp51* background at different ages (n ≥ 9). Columns depict means and error bars represent SEM. (**e**) Direct bilirubin levels in 6-week control, H^*Cyp51*−/−^and runt mice on a wild type or heterozygous *Cyp51* background (n > 10). **p < 0.01; ****p < 0.0001.

From 750 mice, 704 were weaned and a further 46 (6%) exhibited severe developmental abnormalities (runt-H^*Cyp51*−/−^; runts). Runts were exclusive to the H^*Cyp51*−/−^ genotypes. They died or had to be euthanized at 4–10 weeks with a male to female ratio of 2:1 (Supplementary Table 1). Runts are characterized by growth arrest after the weaning period (Fig. 1a), hunched posture, bristled hair and frequent icterus (Fig. 1b). Figures 1c,d show successful *Cyp51* disruption with a progressively increasing gap in *Cyp51* gene and protein levels between H^*Cyp51*−/−^ and control mice. Plasma parameters of these young mice showed mostly unperturbed cholesterol homeostasis, with total (Fig. 2a) and LDL (Fig. 2b) cholesterol remaining stable in H^*Cyp51*−/−^ mice of all examined ages, except for the runts that show a 40% decrease in total cholesterol and a 60% drop in HDL cholesterol (Fig. 2c). Increased ALT but not AST was found in 9-week H^*Cyp51*−/−^ mice and runts, which indicated hepatocellular damage, while elevated conjugated bilirubin in runts confirmed jaundice (Fig. 2d,e).

**Figure 3:**
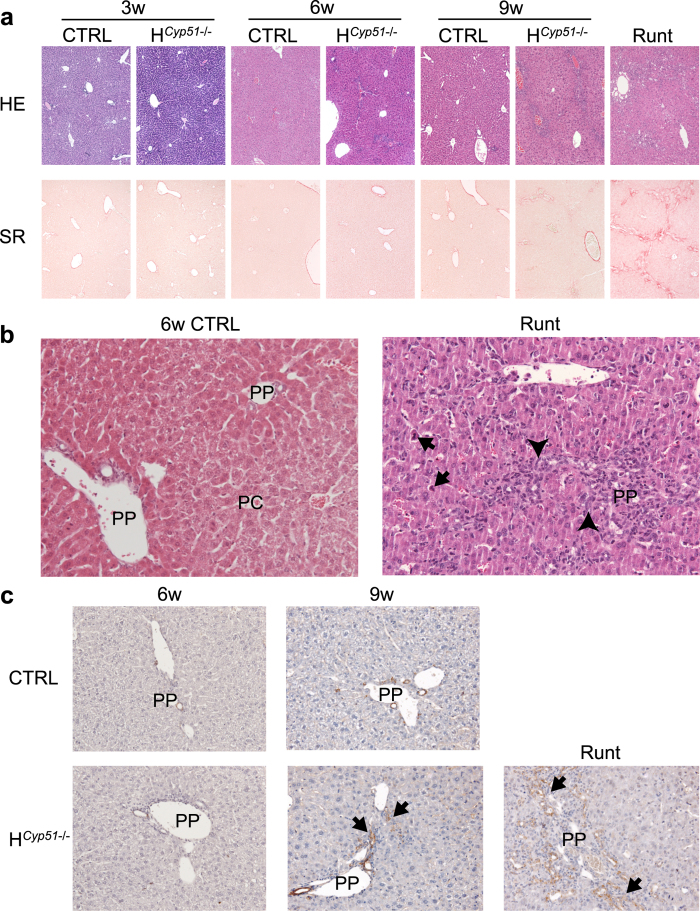
Progressive development of ductular reactions and fibrosis in response to *Cyp51* block in the liver. (**a**) Shown are representative haematoxylin and eosin (top; HE) and Sirius red (bottom; SR) stained liver sections. CTRL – *Cyp51*^+/+^ mice. 100x magnification. (**b**) A representative close up shot of the portal (PP) and central (PC) veins in the 6-week *Cyp51*^+/+^ (CTRL) and the H^*Cyp51*−/−^ runt livers. Arrowheads indicate ductular reaction and arrows apoptotic cells. 200x magnification. (**c**) Shown are representative cytokeratin 19 stained liver sections for 6-week, 9-week and runt mice. Arrows indicate ductular reactions. 200x magnification. CTRL – *Cyp51*^+/+^ mice.

Haematoxylin and eosin (HE) and collagen staining (Sirius red) revealed initial signs of ductular reaction with mild inflammation (lymphocytes and granulocytes) surrounding the portal vein in all H^*Cyp51*−/−^ females (7/7) and about half of males (4/7) at 6 weeks. Enhanced ductular proliferation, inflammation and mild-to-moderate portal fibrosis were seen in all H^*Cyp51*−/−^ mice at 9 weeks (Fig. 3a–c) and were slightly more pronounced on the heterozygous background. Despite their youth, runts exhibited severe liver damage with widespread ductular reaction, immune cell infiltration and prominent fibrosis with portal bridging in the worst cases (Fig. 3b).

### Gene expression profiling reveals progressive metabolic downregulation and a fall in RORC transcriptional potential resulting from *Cyp51* disruption

**Figure 4:**
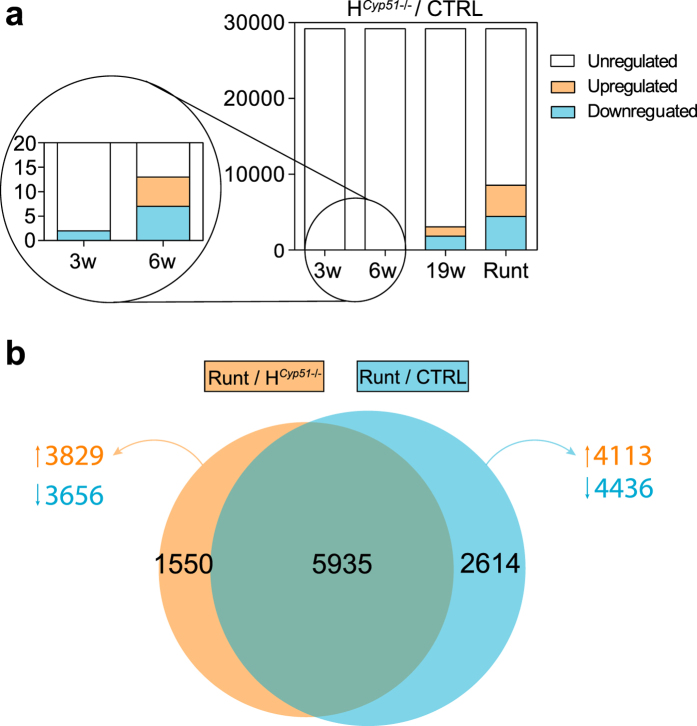
Hepatocyte *Cyp51* disruption leads to progressive metabolic disruption. (**a**) A stacked column plot with numbers of differentially expressed genes comparing H^*Cyp51*−/−^ and control (CTRL) mice on the wild type background for a given age. Runts (5–7 weeks) were compared to 6-week controls. Downregulated genes (blue), upregulated genes (orange), unchanged expression (white). (**b**) Venn diagram comparing the number of DE genes in runts *vs*. 6-week H^*Cyp51*−/−^ mice and runts *vs*. 6-week control mice; all on the wild type *Cyp51* background. The size of the circles correlates to the number of DE genes. The adjacent numbers state the number of corresponding upregulated (orange) or downregulated (blue) DE genes. n = 4 (H^*Cyp51*−/−^; CTRL); n = 6 (runts).

**Figure 5:**
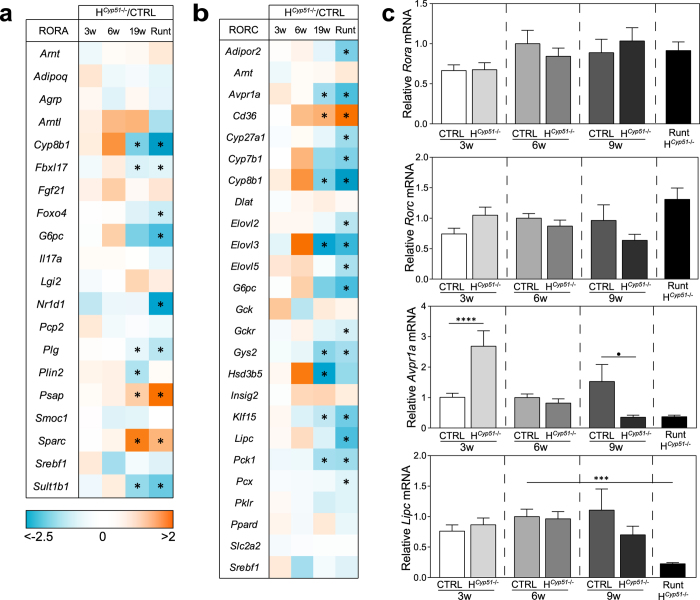
*Cyp51* ablation results in dampened RORA and RORC activities in livers of runt and adult mice. (**a**) A heatmap of RORA target gene expression as measured by microarrays between H^*Cyp51*−/−^ and control (CTRL) mice on the wild type background at different ages. Runts were compared to 6-week controls. Decreased expression (blue); increased expression (orange). n = 4 (H^*Cyp51*−/−^; CTRL); n = 6 (runts). (**b**) A heatmap of RORC target genes as measured by microarrays between H^*Cyp51*−/−^ and control mice on the wild type background at different ages. Runts were compared to 6-week controls. n = 4 (H^*Cyp51*−/−^; CTRL); n = 6 (runts). (**c**) Relative gene expression of *Rora, Rorc* and RORC target genes – *Avpr1a* and *Lipc* – as measured by RT-qPCR in CTRL, H^*Cyp51*−/−^ and runt mice (n > 8), at different ages, on the wild type or heterozygous *Cyp51* background. Columns depict means and error bars represent SEM. ^•^p < 0.1; *p < 0.05; ***p < 0.001; ****p < 0.0001.

**Figure 6:**
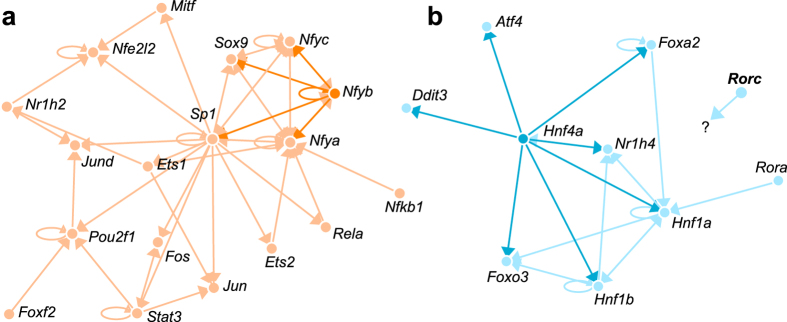
Transcription factor enrichment confirms increased unfolded protein response (UPR) and metabolic decline in runts. (**a**) A network of interactions between selected enriched TFs with increased activity visualized using the Interactome tool. Strong orange colour emphasizes a subcluster of enriched TFs with known involvement in UPR. (**b**) Network of interactions between selected enriched TFs with decreased activity. Dark blue colour designates TFs regulated by HNF4A.

To evaluate the impact of *Cyp51* excision on liver development, we performed gene expression profiling using Affymetrix microarrays on *Cyp51*^+/+^ and H^*Cyp51*−/−^ mice, aged 3, 6 and 19 weeks, of both sexes, including runts. The number of DE genes between H^*Cyp51*−/−^ and control mice increased progressively from 3 weeks onward (Fig. 4a; Supplementary Table 2). Runts exhibited a global de-regulation with roughly a quarter of their hepatic transcriptome differentially expressed compared to 6-week *Cyp51*^+/+^ mice (Fig. 4b). Gene set enrichment analysis (PGSEA) based on pathways from Kyoto Encyclopedia of Genes and Genomes (KEGG) exhibited significant enrichment in runts (235/287 pathways), which are (pre)pubertal mice, and the established 19-week H^*Cyp51*−/−^ mice (202/287 pathways) (Table 1; Supplementary Table 3). Similarly to the 19-week mice (Lorbek *et al*.^8^ and this study), the runt phenotype also associated with downregulated metabolism of lipids, amino acids and bile acids as well as elevated inflammatory and cancer-related pathways, based on their transcriptional profiles (Supplementary Fig. 2). This points to a system-wide de-regulation in gene expression possibly stemming from altered transcriptional landscape. To evaluate this hypothesis, we employed transcription factor (TF) enrichment analysis, which indeed showed significant enrichment in the runt (176/250 TFs) and the adult 19-week H^*Cyp51*−/−^ mice (113/250 TFs) (Table 2; Supplementary Table 4). In both cases RORC and RORA transcriptional activities were among the most downregulated (Fig. 5a–c). Reduced HNF4A activity also indicated metabolic downregulation, while elevated NRF2 and NFY activities indicate that a block in cholesterol synthesis could lead to ROS production^19^ and protein misfolding^20^. The Interactome tool^21^ for visualizing interactions between selected enriched TFs in runts contained no known interactions of RORC with other TFs (Fig. 6), suggesting its diminished activity may stem from other sources, such as the decreased availability of endogenous sterol ligands.

### Sterol imbalance as a major hallmark of *Cyp51* ablation

**Figure 7:**
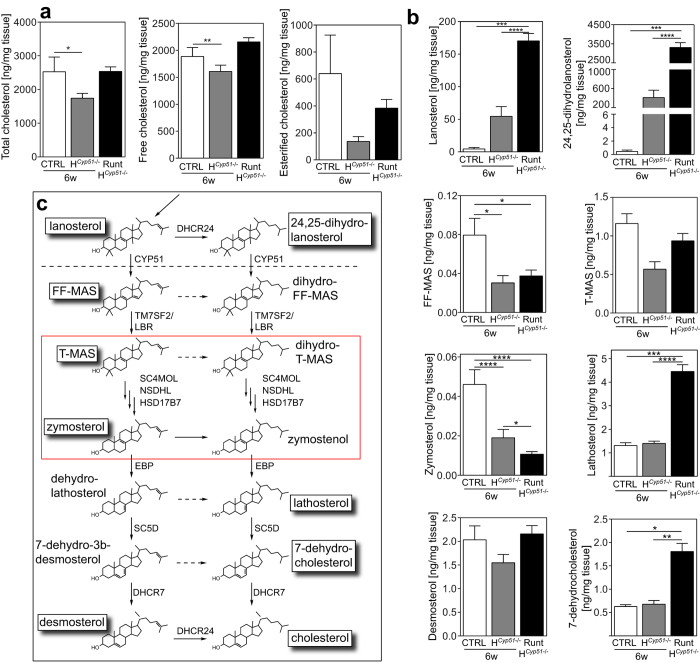
Sterol imbalance as a hallmark of hepatocyte *Cyp51* ablation. (**a**) Liver measurements of total cholesterol (left), free cholesterol (middle) and esterified cholesterol (right) in 6-week control (CTRL), H^*Cyp51*−/−^ and runt males on the wild type *Cyp51* background. Columns depict means and error bars represent SEM (n > 3). Expressed as ng of sterol per mg of wet tissue. (**b**) Levels of selected cholesterol biosynthesis intermediates in livers of 6-week control, H^*Cyp51*−/−^ and runt males on the wild type background. Columns depict means and error bars represent SEM (n > 3). Expressed as ng of sterol per mg of wet tissue. (**c**) Schematic representation of the post-lanosterol part of cholesterol biosynthesis. Red box indicates sterol intermediates proposed as natural RORC ligands. *p < 0.05; **p < 0.01; ***p < 0.001; ****p < 0.0001.

To assess the availability of natural sterol RORC ligands we measured cholesterol and its biosynthesis intermediates by GC/MS. Total cholesterol was diminished in 6-week H^*Cyp51*−/−^ mice, but remained unchanged in runts. This was true also for esterified and free cholesterol (Fig. 7a). CYP51 substrate lanosterol (LAN) was increased 12- and 35-fold and 24,25-dihydrolanosterol (DHL) 800- and 6000-fold in 6-week H^*Cyp51*−/−^ mice and runts, respectively (Fig. 7b). Conversely, the direct product of CYP51 demethylation, follicular fluid meiosis-activating sterol (FF-MAS), was decreased 2.6- and 2.1-fold in 6-week H^*Cyp51*−/−^ mice and runts, respectively. Further downstream the pathway, zymosterol was decreased by 2.4- and 4.3-fold (Fig. 7b). Sterols between MAS and zymosterol (Fig. 7c) were recently proposed as strong RORC agonists^11^. Elevated levels of lathosterol and 7-dehydrocholesterol might support the existence of a shunt pathway, as previously proposed by us^22^,^23^ and others^24^.

### An overlapping transcriptional network of the hepatocyte *Cyp51* and *Rorc* knockout mice

**Figure 8:**
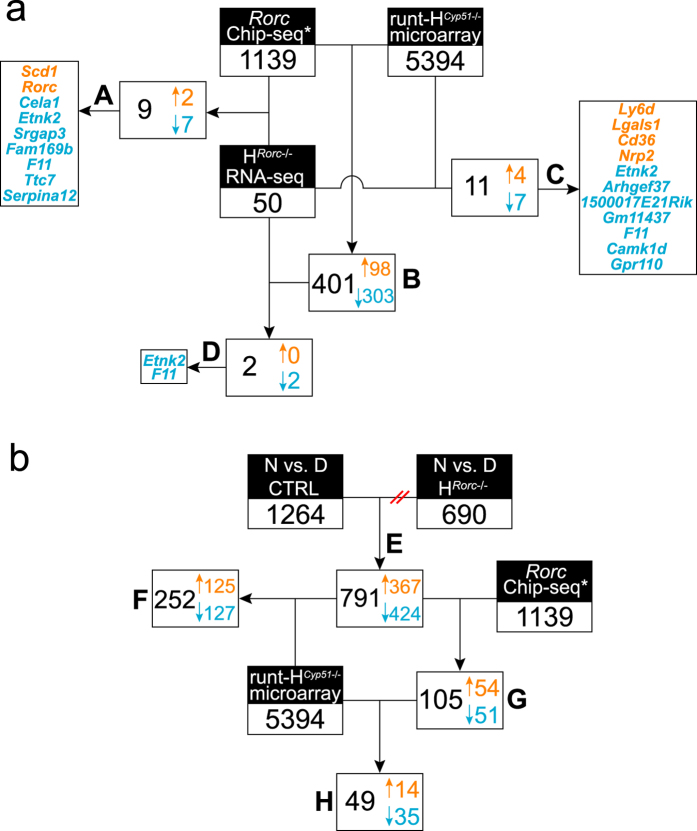
Comparative analysis of H^*Rorc*−/−^ and H^*Cyp51*−/−^ hepatic transcriptomes. (**a**) A three-way comparison of DE genes between H^*Cyp51*−/−^ runts, H^*Rorc*−/−^ mice and chromatin immunoprecipitation (Chip-seq) data for RORC obtained from Takeda *et al*.^17^. A – RORC target genes differentially expressed between H^*Rorc*−/−^ and control mice at ZT7; B - RORC target genes differentially expressed in runts on the wild type background; C – genes differentially expressed in runts and H^*Rorc*−/−^ mice compared to their respective controls; D – RORC target genes differentially expressed in runts and in H^*Rorc*−/−^ mice; (**b**) A sequential comparison of genes regulated by RORC in a diurnal manner with RORC Chip-seq data and data from H^*Cyp51*−/−^ runts. E – genes that showed diurnal (day-night) variation in control (CTRL) mice but not in H^*Rorc*−/−^ mice; F – genes diurnally regulated by RORC that were DE in runts; G – genes diurnally regulated by RORC and identified as RORC targets by Chip-seq analysis (potential RORC targets); H - potential RORC targets differentially expressed in runts. Orange numbers indicate upregulated genes; blue downregulated.

We next set out to evaluate the contribution of diminished RORC activity on the phenotype of H^*Cyp51*−/−^ mice. We applied the hepatocyte *Rorc* (H^*Rorc*−/−^) mice and compared the transcriptome data between the two models. We found 50 DE genes separating H^*Rorc*−/−^ and control mice (Supplementary Table 2), where 11 had the same response as in runts (Fig. 8a, Supplementary Table 5) and 5 as in 19-week H^*Cyp51*−/−^ mice. Due to the known circadian nature of RORC, hepatocytes from the H^*Rorc*−/−^ mice were isolated at ZT7 (daytime RORC nadir) and ZT19 (night-time RORC zenith). TF enrichment of the transcriptome data expectedly showed increased night-time RORC activity only in the control mice since its (circadian) expression is dampened in H^*Rorc*−/−^ livers. From 1264 diurnal DE genes in control mice, 791 lost their rhythm due to RORC abolishment in the H^*Rorc*−/−^ mice and may be considered as potential RORC targets. Moreover, 105 genes from this list were positive for RORC binding based on Chip-seq results by Takeda *et al*.^17^ and a further 49 differentially expressed (mostly downregulated) in runts (Fig. 8b, Supplementary Table 5). FIDEA^25^ showed that these genes are primarily involved amino acid metabolism (Supplementary Tables 6 and 7).

### Age-dependent sexual dimorphism in *Cyp51* ^−/−^ mice

The progressive increase in the number of DE genes between males and females (Supplementary Table 2) is in agreement with the current understanding that sex differences emerge from 4 weeks onward^1^. The number of DE genes in response to *Cyp51* disruption was consistently higher in H^*Cyp51*−/−^ females compared to the males (61/1 at 6 weeks; 1821/941 at 19 weeks). The initial response in 6-week H^*Cyp51*−/−^ females favoured apoptosis and ErbB signalling, pointing to enhanced liver damage and regeneration. At 19 weeks, decreased fatty and amino acid metabolism and enhanced inflammatory and cancer-related pathways were also underlined (Supplementary Table 3). Male sex seems to confer a higher risk for development of the disease phenotype (M:F = 2:1) only in runts. Despite this, runt males and females differed in only 23 genes that showed little to no sex-variation at other ages. The elevation of *Casp12* indicating ER stress and activated unfolded protein response (UPR) represents a hallmark of male runts. TF enrichment also showed elevated activity of NFYs (p = 0.06) and other damage response factors (e.g. JUN, FOS). KEGG pathway enrichment showed better preservation of metabolism and milder inflammation in runt females.

Expression of RORA and RORC is sexually dimorphic with RORC being generally higher in males and RORA in females^26^. The fall in transcriptional activity of both receptors was significantly higher in 19-week H^*Cyp51*−/−^ females, which correlates well with their reduced amino and fatty acid metabolism. These data further implicate ROR signalling as one of the sex-dependent metabolic regulators.

## Discussion

While a major part of cholesterol synthesis is carried out by the liver, other organs contribute to the whole-body cholesterol pool resulting in viable phenotypes of the liver conditional knockouts. Histological and transcriptional changes in H^*Cyp51*−/−^ mice became visible at 6 weeks of age (Supplementary Fig. 3) likely due to cholesterol compensation during the initial 3-week nursing period. The switch to standard laboratory chow lacking cholesterol^27^ raises the need for its endogenous synthesis. As this is disrupted in the knockout livers, the result is a fall in plasma and liver cholesterol at 6 weeks that is remedied at later ages, and alterations of sterol metabolites. Surprisingly, nursing was not always able to prevent liver failure, as indicated by the appearance of H^*Cyp51*−/−^ runts. Runts exhibited the strongest fall in *Cyp51* levels. While the stability of *Cyp51* mRNA and protein is estimated to be in line with other members of the cytochrome P450 family^28^,^29^, the elevated UPR in runts could lead to CYP51 protein misfolding and degradation. Additionally, increased removal of damaged hepatocytes and higher numbers of inflammatory cells that do not express *Cyp51* could have contributed to these measurements. Liver damage in runts most likely started prior to weaning, as witnessed by one 3-week runt-like female. The normal hepatic cholesterol in runts could be explained by the steep fall in plasma HDL. Also based on the sterol profile of DHL we speculate that cholesterol synthesis insufficiency is most pronounced in runts.

From a structural standpoint, cholesterol is an important mediator of membrane fluidity and as a constituent of lipid rafts contributes to cellular signalling. Excess cellular cholesterol preferentially redistributes to intracellular compartments^30^, such as ER, where a raise in cholesterol can trigger UPR and apoptosis in macrophages^31^,^32^. Diminished cholesterol or its replacement with sterol intermediates may also interfere with membrane structure and function^33^,^34^,^35^, demonstrating the need for unperturbed cholesterol homeostasis. Due to methyl groups at carbon C4, cholesterol intermediates LAN and DHL cannot maintain the optimal membrane structure^33^,^36^. Livers of H^*Cyp51*−/−^ mice contained markedly elevated DHL that in runts even surpassed cholesterol. Accumulating DHL could interfere with membranes of mitochondria to produce ROS, with ER to hinder protein synthesis, and with peroxisomes to interrupt metabolic pathways. This is exemplified by activated inflammatory pathways, enrichment of several UPR genes (*Casp12, Eif2ak3*) and TFs (NFY), and decreased peroxisomal metabolism in 19-week H^*Cyp51*−/−^ and runt mice. The steep rise in DHL might therefore represent an initiator of hepatocellular stress and liver damage (Supplementary Fig. 4).

An interesting question is why is there such a large increase in DHL in runts as opposed to the other H^*Cyp51*−/−^ knockouts. In cell cultures, blocking CYP51 resulted in comprehensive LAN and DHL build-up with an almost complete replacement of cellular cholesterol^37^,^38^. Hepatic cholesterol intermediates (including LAN) generally correlate with their plasma concentration, but not in the case of DHL^24^. This suggested that DHL might be metabolized in a shunt pathway as most recently confirmed by us^39^, or might be exported to the bile, which still awaits experimental confirmation. The former option is enticing as it offers an explanation as to why we found no decrease in sterol intermediates immediately before their conversion to cholesterol in H^*Cyp51*−/−^ mice (e.g. desmosterol, 7-dehydrocholesterol). Conversion of DHL to one of the downstream intermediates from the Kandutsch-Russell branch of cholesterol synthesis could also explain the paradoxically increased lathosterol and 7-dehydrocholesterol in runts. Recently, it was also discovered that cholesterol biosynthesis intermediates act as RORγt ligands in Th17 cells^11^,^12^. Based on the current available evidence LAN and DHL could not serve as RORC agonists since a marked increase in RORC activity was observed upon *Cyp51* overexpression that metabolizes both sterols^11^. In contrast, sterols downstream of LAN and DHL in the canonical pathway, as well as non-canonical sterols, such as metabolites of zymosterol, showed specific agonist activity for RORC.

Reduced availability of sterols after the CYP51 block in livers of 19-week H^*Cyp51*−/−^ and runt mice resulted in diminished RORC activity that could contribute to a fall in the overall metabolic capacity (Supplementary Fig. 4). However, the redundancy of binding to ROR response element (RORE) that enables RORA and REV-ERBs to regulate the overlapping set of target genes^17^,^40^, as well as the overlap in regulation potential of other TFs (PPARs, LXR etc.) make the prediction of RORC-specific effects difficult. By applying H^*Rorc*−/−^ mice and comparing genes that lost diurnality upon *Rorc* ablation with genes that bind RORC in their regulatory regions, we were able to overcome this hurdle and pinpointed to RORC transcriptional targets, half of which are de-regulated also in H^*Cyp51*−/−^ runts. These data show that RORC abolishment, after inactivation of *Cyp51*, exerts its effects either by acting directly on gene expression, or by disrupting the normal circadian rhythmicity of metabolic pathways.

With the exception of runts, female H^*Cyp51*−/−^ mice exhibited a more prominent liver phenotype compared to males throughout their development. This may attribute to the reduced production of oestrogens with antioxidant and anti-fibrotic properties^41^, as well as to the diminished amino acid and lipid metabolism, corresponding to a stronger fall in RORA and RORC activities in 19-week H^*Cyp51*−/−^ females. Conversely, higher prevalence of runt males with dampened metabolism and increased UPR points to pre-pubertal protection of females. Liver thus responds to cholesterol synthesis block in a sex- and age-dependent manner. This is exemplified also in the hepatocyte *Hmgcr*^−/−^ mice^7^, that similarly to H^*Cyp51*−/−^ runts, died by 5 weeks, with minor changes in plasma and liver cholesterol and an activated ER stress response. While the hepatocyte *Hmgcr*^−/−^ knockout was lethal in males, 30% of females survived for up to a year, reflecting a similar sexual dimorphism as observed in H^*Cyp51*−/−^ runts.

In summary, we provide evidence that cholesterol synthesis disruption reduces RORC activity *in vivo*, likely due to the lack of endogenous sterol ligands. We also show that during development towards adulthood female and male livers differ in their susceptibility to the cholesterol synthesis block, where dampened ROR signalling and decreased metabolism likely contribute to this progressive liver disease.

## Methods

### Ethics Statement

Mice experiments were performed in compliance with the current guidelines and regulations. In the case of *Cyp51* mice, they were approved by the Veterinary Administration of the Republic of Slovenia (license numbers 34401-31/2011/4 and 34401-52/2012/3) and were conducted in agreement with the European Convention for the Protection of Vertebrate Animals used for Experimental and Other Scientific Purposes (ETS 123) as well as with the guidelines for work with laboratory animals by the National Institute of Health. In the case of *Rorc* mice, experiments were performed in accordance with protocols approved by the Institutional Animal Care and Use Committee of the NYU School of Medicine.

### Animals

Hepatocyte *Cyp51* knockout mice were prepared by crossbreeding *Cyp51*^*flox/flox*^ or *Cyp51*^*-/flox*^ with *Alb-Cre* mice to generate hepatocyte specific *Cyp51*^−/−^ mice on a wild type (*Cyp51*^*flox/flox*^ *Alb-Cre;* designated as *Cyp51*^+/+^ H^*Cyp51*−/−^) and heterozygous (*Cyp51*^*-/flox*^ *Alb-Cre;* designated as *Cyp51*^+/−^ H^*Cyp51*−/−^) background^8^,^42^. *Cyp51*^*flox/flox*^ (*Cyp51*^+/+^) and heterozygous *Cyp51*^*-/flox*^ (*Cyp51*^+/−^) mice were used as controls^27^. Mice were euthanized at different time points during development – 0, 3, 6, 9 and 19 weeks. Blood and liver were collected for biochemical and histological analyses.

Hepatocyte *Rorc* knockout mice (H^*Rorc*−/−^) were prepared by crossbreeding *Rorc*^*flox/flox*^ and *Alb-Cre* mice. Hepatocytes were isolated from 12–13 week old mice at ZT7 and ZT19 using the two-step collagenase perfusion method.

### Histology

Formalin-fixed and paraffin-embedded liver sections were cut at 5 μm and stained with hematoxylin and eosin for general histological evaluation or with Sirius red to assess collagen deposition.

### Biochemical analyses

Plasma parameters (cholesterol, ALT, AST, bilirubin) were measured using commercially available kits. Liver sterols were evaluated by coupled gas spectrometry/mass spectrometry as previously described^43^. Total proteins and RNA was isolated from frozen liver samples.

### Microarray-based gene expression profiling

Affymetrix GeneChip® Mouse Gene 2.0 ST arrays were used to conduct gene expression profiling on *Cyp51*^+/+^ and *Cyp51*^+/+^ H^*Cyp51*−/−^ mice of both sexes, aged 3, 6, 19 weeks and runts (30 samples). Gene expression analysis was done in R using Bioconductor packages limma and PGSEA. Gene sets for enrichment analysis were taken from the KEGG and TRANSFAC databases.

### RNA Sequencing

Libraries were prepared using the Illumina TruSeq Stranded Total RNA library prep, with Ribozero Gold, starting from 500 ng of total RNA and loaded on high output HiSeq 2500 flow cells. RNA alignments were performed using STAR and reads mapping to exons counted using featureCounts. Counts were normalized in R using the DESeq2 package.

Detailed protocols can be found in the supplementary information file.

## Additional Information

**How to cite this article:** Urlep, Z. *et al*. Disrupting Hepatocyte *Cyp51* from Cholesterol Synthesis Leads to Progressive Liver Injury in the Developing Mouse and Decreases RORC Signalling. *Sci. Rep.* **7**, 40775; doi: 10.1038/srep40775 (2017).

**Publisher's note:** Springer Nature remains neutral with regard to jurisdictional claims in published maps and institutional affiliations.

## Supplementary Material

Supplementary Information

Supplementary Table 2

Supplementary Table 3

Supplementary Table 4

Supplementary Table 5

## Figures and Tables

**Table 1:** Selected upregulated and downregulated enriched KEGG pathways in runt-H^*Cyp51*−/−^ mice on the wild type background compared to 6-week *Cyp51* ^+/+^ mice.

KEGG pathway	DE genes/all genes in a pathway	FDR p-value
Upregulated		
TNF signalling pathway	50/107	<0.001
NF-kappa B signalling pathway	49/84	<0.001
Focal adhesion	108/206	<0.001
Apoptosis	49/80	<0.001
Chemokine signalling pathway	91/174	<0.001
Downregulated		
Peroxisome	61/81	<0.001
Nitrogen metabolism	8/17	<0.001
Primary bile acid biosynthesis	14/16	<0.001
Valine, leucine and isoleucine degradation	36/54	<0.001
Steroid biosynthesis	7/20	<0.05

**Table 2:** Selected upregulated and downregulated enriched TFs in runt-H^*Cyp51*−/−^ mice on the wild type background compared to 6-week *Cyp51* ^+/+^ mice.

TF	FDR p-value	Association with phenotype
Upregulated		
*Nfy*	<0.001	Involvement in UPR^20^
*Nrf2*	<0.001	Regulates the response to oxidative stress^44^
*Jun*	<0.001	NAFLD^45^, cancer/proliferation^46^
*Sox9*	<0.001	Ductular reactions^47^
*Rela*	<0.001	Inflammation, regeneration, fibrosis^48^
Downregulated		
*Hnf4a*	<0.001	Central metabolic regulator^49^
*Foxa2 (Hnf3b*)	<0.001	Bile acid homeostasis^50^, lipid metabolism^51^, apoptosis^52^
*Rorc*	<0.001	Lipid, cholesterol and glucose metabolism^17^,^18^
*Rora*	<0.001	Lipid metabolism^53^, cholesterol as ligand^54^
*Fxr*	<0.01	Bile acids homeostasis^55^
