# Supplementary material for: Disrupting Hepatocyte Cyp51 from Cholesterol Synthesis Leads to Progressive Liver Injury in the Developing Mouse and Decreases RORC Signalling
Source: Sci Rep. 2017 Jan 18;7:40775. doi: 10.1038/srep40775 (PMC5241696; doi:10.1038/srep40775)
Supplement: Supplementary Information [file srep40775-s1.pdf]

Supplementary information

Disrupting Hepatocyte *Cyp51* from Cholesterol Synthesis Leads to Progressive Liver Injury in the Developing Mouse and Decreases RORC Signalling

Žiga Urlep[1], Gregor Lorbek[1], Martina Perše[2], Jera Jeruc[3], Peter Juvan[1], Madlen Matz-Soja[4], Rolf Gebhardt[4], Ingemar Björkhem[5], Jason A. Hall[6], Richard Bonneau[7], Dan R. Littman[6,8] and Damjana Rozman[1*]

[1]Centre for Functional Genomics and Bio-Chips, Institute of Biochemistry, Faculty of Medicine, University of Ljubljana, Ljubljana, Slovenia

[2]Medical Experimental Centre, Institute of Pathology, Faculty of Medicine, University of Ljubljana, Ljubljana, Slovenia

[3]Institute of Pathology, Faculty of Medicine, University of Ljubljana, Ljubljana, Slovenia

[4]Institute of Biochemistry, Faculty of Medicine, University of Leipzig, Leipzig, Germany

[5]Department of Laboratory Medicine, Division of Clinical Chemistry, Karolinska Institute, Karolinska University Hospital, Huddinge, Sweden

[6]The Kimmel Center for Biology and Medicine of the Skirball Institute, New York University School of Medicine, New York, New York 10016, USA

[7]New York University & Simons Foundation for Data Analysis, New York, NY 10010, USA

[8]Howard Hughes Medical Institute, New York University School of Medicine, New York, New York 10016, USA

Supplementary methods

Animals

The generation of *Cyp51* heterozygous (*Cyp51*$^{-/\mathrm{flox}}$, *Alb-Cre*$^{-}$; designated as *Cyp51*$^{+/-}$) mice and hepatocyte specific *Cyp51* knockout mice (*Cyp51*$^{\mathrm{flox/flox}}$, *Alb-Cre*$^{+}$; designated as *Cyp51*$^{+/+}$ H$^{Cyp51-/-}$) has been reported previously[1-3]. Briefly, *Cyp51*$^{+/-}$ or *Cyp51*$^{+/+}$ (*Cyp51*$^{\mathrm{flox/flox}}$, *Alb-Cre*$^{-}$) mice on a mixed genetic background ((up to 10 %) 129/Pas x (at least 90 %) C57BL/6J) were cross-bred with *Alb-Cre*$^{+}$ mice (B6.Cg-Tg(Alb-cre)21Mgn/J; backcrossed 5 times to the C57BL/6J background) to generate the hepatocyte specific knockouts (H$^{Cyp51-/-}$) on either the wild type (*Cyp51*$^{+/+}$) or the heterozygous (*Cyp51*$^{+/-}$) background. The offspring were genotyped based on gDNA from the tail or ear and the deletion of *Cyp51* exons 3 and 4[1]. Mice were housed at the Medical experimental center's animal facility under controlled conditions – temperature of 22±1 ºC, humidity 55±10% and 12:12-hour light–dark cycle (7:00–19:00 light) with unlimited access to water and diet (Altromin 1324, Germany).
For experiments, the knockout and the control mice were pooled into two groups (H$^{Cyp51-/-}$; CTRL) regardless of the *Cyp51* background (excluding the microarrays experiment, where only mice on the *Cyp51*$^{+/+}$ background were used), due to the absence of significant background effect on *Cyp51* expression and mouse development (Supplementary Fig. 5).
All procedures involving animals were approved by the Administration of the Republic of Slovenia for Food Safety, Veterinary and Plant Protection (Permit No. 4401-31/2011/4 and 34401-52/2012/3). Animal care and treatment were conducted in accordance with the national legislation and international laws and guidelines (Directive 2010/63/EU on the protection of animals used for scientific purposes, National Institute of Health guidelines for work with laboratory animals).

For the generation of hepatocyte specific *Rorc* knockouts (*Rorc*$^{flox/flox}$, *Alb-Cre*$^{+}$; H$^{Rorc-/-}$), C57BL/6 mice carrying floxed alleles at *Rorc* loci were crossed with *Alb-Cre* transgenic mice purchased from Jax laboratories. Animals were bred and maintained under a 12:12-hour light–dark cycle (06:30–18:30 light) in the animal facility of the Skirball Institute (NYU School of Medicine) in specific-pathogen free (SPF) conditions. All experiments on mice were performed in accordance with protocols approved by the Institutional Animal Care and Use Committee of the NYU School of Medicine.

Sample collection

To evaluate the phenotype of the H$^{Cyp51-/-}$ mice, clinical picture was monitored and recorded on a daily basis. Mice were euthanized by cervical dislocation between 7:30 and 13:30 at the specified ages – at birth (day 0), weaning (3 weeks), puberty (6 weeks) and following sexual maturity (9 and 19 weeks), as recommended by the Working document on genetically altered animals. At autopsy blood was taken by a heart puncture and internal organs (liver, kidney, spleen, heart, gonads) were weighed and macroscopically examined. Plasma was collected into heparin coated Vacuette MiniCollect® 1 ml Plasma Tubes (Greiner Bio-one, Frickenhausen, Germany) and organs were snap-frozen in liquid nitrogen. Left lateral liver lobes were fixed in formalin and embedded in paraffin for further histological analyses.
In case of runt- H$^{Cyp51-/-}$ mice, humane end points were set at which they were euthanized and their organs were taken for further analyses, as described above.
Hepatocytes from 12-13 week old male H$^{Rorc-/-}$ or littermate *Rorc*$^{flox/flox}$, *Alb-Cre*$^{-}$ (control) mice were harvested by the two-step collagenase perfusion method (described below) at ZT7 or ZT19.

Collagenase perfusion

Following anesthetization of mice with isoflurane, an intraperitoneal incision was made to expose the portal vein and vena cava. A 24-gauge perfusion needle (BD Nexiva 24 GA IV catheter) was then inserted into the portal vein. Once secured, the vena cava was cut and perfusion of the liver was performed. Perfusion was carried out with, 1) chelating solution (0.5 mM EGTA and 0.05 M Hepes) for 5 min. Flow from the vena cava was intermittently blocked to ensure correct needle insertion. Mice were subsequently switched to, 2) digestion solution (5% fetal calf serum, 1.0 mg/mL of Collagenase D (Roche), 100 μg/mL DNase I (Sigma), 0.1 U/mL Dispase (Worthington), 4.8 mM CaCl2, and 0.05 M Hepes) for 5 min. Each solution was prepared in Ca/Mg free HBSS (GIBCO) and degassed prior to being administered at a flow rate of 10mL/min from a water bath maintained at 42 °C. Upon digestion, the liver was carefully excised and teased apart atop a 100 μm filter. William's E medium (GIBCO) supplemented with 2% FCS and 2 mM glutaMAX (GIBCO) was then passed over the tissue while swirling the tip of a pipet to further break apart the tissue. Cells were centrifuged at 50 g for 4 min to prevent the collection of dead cells and non-hepatocytes and washed 3x in William's E medium. Cells were resuspended in Trizol (Life Technologies), passed through a 20-gauge needle to create a homogenate and flash frozen to store RNA.

Histological analysis

Paraffin embedded liver was sectioned to 5 μm on glass slides and stained with haematoxylin and eosin (HE) for general histological assessment or with Sirius red (SR; 0,1 % direct red 80, 1,2 % picric acid in water) to evaluate the degree of fibrosis.

HE staining was done at the Institute for Pathology, Medical Faculty, University of Ljubljana. Samples were checked for hepatocyte morphology and viability (apoptosis, mitosis), presence of steatosis, infiltration and localization of immune cells and ductular abnormalities.

For SR staining, samples were deparaffinized by incubating at 70 °C for 10 min and sequential washing in Xylene (2x), 100% ethanol, 95% ethanol, 70% ethanol and water (2x). Glass slides were incubated with SR solution for 1 h and briefly destained in diluted acetic acid (10 mL concentrated acetic acid in 22 mL water). Afterwards, samples were dehydrated sequential washing in 70 % ethanol, 95 % ethanol, 100 % ethanol and Xylene (2x) and fixed with Roti Histokitt II (Carl Roth GmbH + Co. KG, Germany). Stained samples were qualitatively evaluated for the degree of fibrosis (score of 0 to 3, with 0 being absent and 3 being very strong with bridging).

Immunohistochemistry

Paraffin embedded liver was sectioned to 4 µm on glass slides. Samples were deparaffinised and rehydrated, followed by heat-induced epitope retrieval in 0.01 M citrate buffer (pH = 6) for 20 min in a 900 W microwave oven. After cooling, samples were incubated in 3% H_2O_2 in TBS at room temperature (RT) for 10 min to block endogenous peroxidases and in 5% goat serum (G9023, Sigma-Aldrich, St. Louis, MO, USA) for 1h at RT to prevent unspecific antibody binding. To stain cholangiocytes, we used primary rabbit anti-cytokeratin 19 antibodies (LS Bioscience LS-B5801; dilution 1:500) in 1 % goat serum in 0.1% TBST and incubated overnight at 4°C. For detection, we used the DAKO EnVision Detection System (K5007, Agilent Technologies DAKO, Glostrup, Denmark) according to the manufacturer's instructions.

RNA isolation and cDNA synthesis

Total RNA from *Cyp51*$^{+/+}$ and H$^{Cyp51-/-}$ mice was isolated using Fuji QuickGene-810 (Fujifilm, Singapore) with the QuickGene Tissue Kit S II according to the manufacturer's instructions from cca. 30 mg of frozen liver tissue. RNEasy Mini Kit (Qiagen) was used in the case of RNA isolation from H$^{Rorc-/-}$ and control mice. RNA concentration was measured using NanoDrop 1000 spectrophotometer (Thermo Fischer Scientific, Waltham, MA, USA) and quality was checked with Agilent 2100 BioAnalyzer (Agilent Technologies, Santa Clara, CA, USA). Prior to reverse transcription, RNA was subjected to treatment with DNase I (Roche, Basel, Switzerland) to limit DNA contamination. Reverse transcription was done from 2 µg of RNA with Transcriptor Universal cDNA Master (Roche, Basel, Switzerland) according to manufacturer's instructions.

Gene expression analysis by qPCR

Gene expression was measured by quantitative reverse transcription polymerase chain reaction (qRT-PCR) using Roche LightCycler 480 (Roche, Basel, Switzerland). A minimum of 3 samples per group were used and for each sample 3 technical replicas were made. The PCR reaction contained: 0.75 µL of cDNA, 0.6 µL of primer mix (2.5 µM concentration of each primer), 2.5 µL of Sybr Green I Master (Roche, Basel, Switzerland) and 1.15 µL of PCR grade water, to a final volume of 5 µL per reaction. The thermocycling program consisted of: 10 min incubation at 95 °C; 45 cycles of 10 s at 95 °C, 20 s at 60 °C and 20 s at 72 °C. Relative expression was calculated as previously described[4]. *Hmbs* and *Ppib* were chosen as reference genes for normalization by using the NormFinder[5] and BestKeeper[6] algorithms. Primer sequences are listed in a separate table (Supplementary Table 8). One-way ANOVA was used for analysing data with only one relevant factor. Two-way ANOVA

was used to evaluate the effects of multiple factors. Holm-Sidak correction for multiple comparisons was used and a p value threshold of 0.05 was used as a measurement of significance.

Protein isolation and Western blot analysis

Total proteins were isolated using lysis buffer (20 mM Tris/HCl pH 7.5, 150 mM NaCl, 1% NP-40, 5 mM EDTA) containing 1 mM PMSF and cOmplete protease inhibitor cocktail (Roche, Basel, Switzerland). Approximately 30 mg of tissue was homogenized in complete lysis buffer and incubated for 2 h at 4°C. Following centrifugation at 12,000 g, 4°C for 15 min, protein concentration was measured using Pierce™ BCA Protein Assay Kit (Thermo Fischer Scientific, Waltham, MA, USA) according to manufacturer's instructions.

Samples were pooled together in equal amounts based on their age, sex and genotype (except for 9-week $H^{Cyp51-/-}$ females, where only 1 sample was obtained) and mixed with NuPAGE® LDS Sample Buffer (Thermo Fischer Scientific, Waltham, MA, USA) at a ratio of 1:3. Samples (10 µg per sample pool) were separated on a 12% SDS-PAGE gel and transferred to a PVDF membrane (Immobilon-P Membranes, Millipore, Billerica, MA, USA). The membrane was blocked using 5% (w/V) non-fat dry milk in PBST (0.1% Tween 20) for 1 h at room temperature. Next followed the incubation with anti-mouse-CYP51 antibodies (self-made rabbit polyclonal antibody against peptide QRLKDSWAERLDFNPDRY; 1:250) and subsequently with peroxidase conjugated goat anti-rabbit antibodies (A0545, Sigma-Aldrich, St. Louis, MO, USA; 1:10000). Visualization was done with SuperSignal™ West Pico Chemiluminescent Substrate (Thermo Fischer Scientific, Waltham, MA, USA) and chemiluminescence recorded using LAS-4000 camera (Fujifilm,

Singapore). To control for protein loading, membranes were stained with Ponceau S solution.

Plasma measurements

Total, HDL and LDL cholesterol, alanine aminotransferase (ALT), aspartate aminotransferase (AST), direct and total bilirubin were analysed by Veterinarska ambulanta BTC (Ljubljana, Slovenia) with Architect ci8200 analyser (Abbott Diagnostics, Abbott Park, IL, USA), >5 samples per group. Concentrations of lipid parameters are given in mmol/L, direct bilirubin in µmol/L and the activity of aminotransferases was measured as µkat/L. Two-way ANOVA was used to evaluate the effects of multiple factors (e.g. age and genotype). Holm-Sidak correction for multiple comparisons was used and a p-value threshold of 0.05 was used as a measurement of significance.

Total sterol extraction and GC/MS analysis

Sterols extraction from frozen liver and coupled gas chromatography/mass spectrometry (GC/MS) analysis were conducted as previously described[7, 8]. A minimum of 3 samples per group were analysed. Sterol amounts are expressed as ng of compound per mg of wet liver tissue. One-way ANOVA was used to analysing the impact of one factor (e.g. genotype) and two-way ANOVA for multiple factors (e.g. age and genotype). Holm-Sidak correction for multiple comparisons was used and a p-value threshold of 0.05 was used as a measurement of significance.

Microarray-based gene expression profiling

We used 30 Affymetrix GeneChip® Mouse Gene 2.0 ST Arrays (Affymetrix, Santa Clara, CA, USA), with 2 chips per group (age-sex-genotype) in the case of control and $H^{Cyp51-/-}$ mice on the wild type background and 3 in the case of runts on the wild

type background. The experiment was performed as previously described [3]. Briefly, 250 ng of starting RNA with a RIN > 8.0 was reverse transcribed as per manufacturer's instructions. After a 16 h hybridization step at 45°C, arrays were washed and stained on GeneChip Fluidics Station 450 and subsequently scanned on Affymetrix GeneChip Scanner 3000 7G. Image analysis and quality check were done using Affymetrix Expression Console™ version 1.3.

Quality check and gene expression analysis were done using R and Bioconductor packages. Quality control and RMA-based normalization of gene expression data were performed using xps package[9]. Raw (CEL) as well as normalized data were deposited to GEO under accession number GSE78892. Package limma[10] was used to infer differential expression of genes and enrichment of gene sets using three predictor variables (age, sex and genotype) and their interactions (age x genotype, age x sex, sex x genotype). Gene sets were constructed using KEGG pathways[11] and TRANSFAC database[12]. Sets containing over 5 elements were tested for enrichment using the PGSEA package[13]. In the case of transcription factor enrichment, factors were merged based on their ID irrespective of their binding sites. For RORC and RORA, target genes were updated based on the literature[14-16] (RORalpha – original gene set based on TRANSFAC; Rora – updated gene set). False discovery rate (FDR) was used to account for multiple hypothesis testing. Significance level $\alpha = 0.05$ was used to control the rate of Type I error for the differential gene expression as well as for the pathway enrichment. The network diagram (Supplementary Fig. 3) was created using Cytoscape program[17] and ClueGo plugin[18]. Analysis of KEGG pathways with kappa score set to 0.2. Gene set enrichment on the proposed RORC target genes was conducted using the FIDEA tool[19]. The Interactome tool[20, 21] was used on selected up- or downregulated

enriched transcription factors to evaluate their interaction and identify central nodes of regulation.

Library construction and sequencing

RNASeq libraries were prepared using the Illumina TruSeq Stranded Total RNA library prep, with Ribozero Gold, starting from 500 ng of total RNA, following the manufacturer's protocol, with the exception that 13 cycles of PCR were performed to amplify the libraries, to keep the duplication rate lower than with the recommended 15 cycles. The amplified libraries were purified using AMPure beads, quantified by Qubit and QPCR, and visualized in an Agilent Bioanalyzer. The libraries were pooled equimolarly, and loaded at 8 pM, on high output HiSeq 2500 flow cells, using v4 reagents, as paired 50 nucleotide reads. Libraries were pooled and distributed uniformly across 3 lanes in order to generate 60-80 million reads per sample.

RNA-Seq Analysis

RNA-seq alignments were performed using STAR (v2.4.2a modified)[22]. Alignments were filtered to remove those having a quality score less than 30; subsequently reads mapping to exons were counted using featureCounts (v1.4.6)[23] and summarized by gene. TDF views (IGV v2.3.32)[24] were generated from the quality filtered alignments. The UCSC genes annotation (May 23, 2014)[25], and primary assembly (Dec. 2011 GRCm38/mm10)[26], for mm10 were obtained from iGenomes (https://support.illumina.com/sequencing/sequencing_software/igenome.html), and were used respectively as annotation and reference genome. Where parameters are not explicitly stated below, defaults of the specified software versions were applied. DESeq2[27] for R was used to normalize the counts and infer gene differential expression.

PARAMETERS:

STAR
--outFilterType BySJout
--outFilterMultimapNmax 20
--alignSJoverhangMin 8
--alignSJDBoverhangMin 1
--outFilterMismatchNmax 999
--outFilterMismatchNoverLmax 0.04
--alignIntronMin 20
--alignIntronMax 1000000
--alignMatesGapMax 1000000
featureCounts
-t exon
-g gene_id

Supplementary references

1. Keber, R. et al. Mouse knockout of the cholesterogenic cytochrome P450 lanosterol 14alpha-demethylase (Cyp51) resembles Antley-Bixler syndrome. *J Biol Chem* **286**, 29086-29097 (2011).
2. Lewinska, M. et al. Hidden disease susceptibility and sexual dimorphism in the heterozygous knockout of cyp51 from cholesterol synthesis. *PLoS One* **9**, e112787 (2014).
3. Lorbek, G. et al. Lessons from hepatocyte-specific cyp51 knockout mice: impaired cholesterol synthesis leads to oval cell-driven liver injury. *Sci Rep* **5**, 8777 (2015).
4. Vandesompele, J. et al. Accurate normalization of real-time quantitative RT-PCR data by geometric averaging of multiple internal control genes. *Genome Biol* **3**, RESEARCH0034 (2002).
5. Andersen, C.L., Jensen, J.L. & Orntoft, T.F. Normalization of real-time quantitative reverse transcription-PCR data: a model-based variance estimation approach to identify genes suited for normalization, applied to bladder and colon cancer data sets. *Cancer Res* **64**, 5245-5250 (2004).
6. Pfaffl, M.W., Tichopad, A., Prgomet, C. & Neuvians, T.P. Determination of stable housekeeping genes, differentially regulated target genes and sample integrity: BestKeeper--Excel-based tool using pair-wise correlations. *Biotechnol Lett* **26**, 509-515 (2004).
7. Lorbek, G., Perse, M., Horvat, S., Bjorkhem, I. & Rozman, D. Sex differences in the hepatic cholesterol sensing mechanisms in mice. *Molecules* **18**, 11067-11085 (2013).

8. Acimovic, J. et al. Combined gas chromatographic/mass spectrometric analysis of cholesterol precursors and plant sterols in cultured cells. *J Chromatogr B Analyt Technol Biomed Life Sci* **877**, 2081-2086 (2009).

9. Stratowa, C., Edn. R package version 1.30.0 (

10. Smyth, G.K. Linear models and empirical bayes methods for assessing differential expression in microarray experiments. *Stat Appl Genet Mol Biol* **3**, Article3 (2004).

11. Kanehisa, M. & Goto, S. KEGG: kyoto encyclopedia of genes and genomes. *Nucleic Acids Res* **28**, 27-30 (2000).

12. Matys, V. et al. TRANSFAC and its module TRANSCompel: transcriptional gene regulation in eukaryotes. *Nucleic Acids Res* **34**, D108-110 (2006).

13. Furge, K. & Dykema, K., Edn. R package version 1.44.0 (

14. Takeda, Y., Jothi, R., Birault, V. & Jetten, A.M. RORgamma directly regulates the circadian expression of clock genes and downstream targets in vivo. *Nucleic Acids Res* **40**, 8519-8535 (2012).

15. Takeda, Y. et al. Retinoic acid-related orphan receptor gamma (RORgamma): a novel participant in the diurnal regulation of hepatic gluconeogenesis and insulin sensitivity. *PLoS Genet* **10**, e1004331 (2014).

16. Takeda, Y. et al. Retinoid acid-related orphan receptor gamma, RORgamma, participates in diurnal transcriptional regulation of lipid metabolic genes. *Nucleic Acids Res* **42**, 10448-10459 (2014).

17. Shannon, P. et al. Cytoscape: a software environment for integrated models of biomolecular interaction networks. *Genome Res* **13**, 2498-2504 (2003).

18. Bindea, G. et al. ClueGO: a Cytoscape plug-in to decipher functionally grouped gene ontology and pathway annotation networks. *Bioinformatics* **25**, 1091-1093 (2009).

19. D'Andrea, D., Grassi, L., Mazzapioda, M. & Tramontano, A. FIDEA: a server for the functional interpretation of differential expression analysis. *Nucleic Acids Res* **41**, W84-88 (2013).

20. Neph, S. et al. Circuitry and dynamics of human transcription factor regulatory networks. *Cell* **150**, 1274-1286 (2012).

21. Stergachis, A.B. et al. Conservation of trans-acting circuitry during mammalian regulatory evolution. *Nature* **515**, 365-370 (2014).

22. Dobin, A. et al. STAR: ultrafast universal RNA-seq aligner. *Bioinformatics* **29**, 15-21 (2013).

23. Liao, Y., Smyth, G.K. & Shi, W. featureCounts: an efficient general purpose program for assigning sequence reads to genomic features. *Bioinformatics* **30**, 923-930 (2014).

24. Robinson, J.T. et al. Integrative genomics viewer. *Nature biotechnology* **29**, 24-26 (2011).

25. Meyer, L.R. et al. The UCSC Genome Browser database: extensions and updates 2013. *Nucleic Acids Res* **41**, D64-69 (2013).

26. Mouse Genome Sequencing, C. et al. Initial sequencing and comparative analysis of the mouse genome. *Nature* **420**, 520-562 (2002).

27. Love, M.I., Huber, W. & Anders, S. Moderated estimation of fold change and dispersion for RNA-seq data with DESeq2. *Genome Biol* **15**, 550 (2014).

Supplementary Table 1: The number of mice based on sex and genotype, with number of runts per group shown in brackets and percentage of runts per group in bold.

	**Genotype**						
**Gender**	$Cyp51^{+/+}$	$Cyp51^{+/-}$	**Controls**	$Cyp51^{+/+}$ $H^{Cyp51-/-}$	$Cyp51^{+/-}$ $H^{Cyp51-/-}$	**$H^{Cyp51-/-}$**	
Female	101 (0)<br>0 %	83 (0)<br>0 %	**184 (0)<br>0 %**	79 (6)<br>5 %	74 (9)<br>3 %	**153 (15)<br>10 %**	
Male	97 (0)<br>0 %	79 (0)<br>0 %	**176 (0)<br>0 %**	99 (16)<br>15 %	92 (15)<br>11 %	**191 (31)<br>16 %**	
Total	198 (0)<br>0 %	162 (0)<br>0 %	**360 (0)<br>0 %**	178 (25)<br>14 %	166 (24)<br>14 %	**344 (46)<br>13 %**	**704<br>(46)<br>7 %**

Supplementary Table 2: A list of differentially expressed genes with the corresponding log-fold change (logFC) and adjusted p-values comparing $H^{Cyp51-/-}$ and control mice on the *Cyp51*$^{+/+}$ background by genotype or by sex at 3, 6 and 19 weeks of age. Runt mice are compared to 6-week *Cyp51*$^{+/+}$ mice. $H^{Rorc-/-}$ mice were compared with controls based on genotype or based on the isolation time (ZT7=day; ZT19=night). An empty list signifies that no differentially expressed genes were found for the selected comparison.

Supplementary Table 3: A list of enriched KEGG pathways with the corresponding logFC and adjusted p-values comparing $H^{Cyp51-/-}$ and control mice on the *Cyp51*$^{+/+}$ background by genotype or by sex at 3, 6 and 19 weeks of age. Runt mice were compared to 6-week *Cyp51*$^{+/+}$ mice. $H^{Rorc-/-}$ mice are compared with controls based on genotype or based on the isolation time (ZT7=day; ZT19=night). An empty list signifies that no enriched pathways were found for the selected comparison.

Supplementary Table 4: A list of enriched transcription factors with the corresponding logFC and adjusted p-values comparing $H^{Cyp51-/-}$ and control mice on the *Cyp51*$^{+/+}$ background by genotype or by sex at 3, 6 and 19 weeks of age. Runt mice were compared to 6-week *Cyp51*$^{+/+}$ mice. $H^{Rorc-/-}$ mice are compared with controls based on genotype or based on the isolation time (ZT7=day; ZT19=night). An empty list signifies that no enriched transcription factors were found for the selected comparison.

Supplementary Table 5: Overlapping differentially expressed genes ($p<0.05$) for comparisons A-H based on Figure 8 with appropriate fold-change values.

Supplementary Table 6: KEGG pathway enrichment for 49 RORC target genes that were also DE in H$^{Cyp51-/-}$ runts.

ID_CATEGORY	NAME_CATEGORY	ADJUSTED_PVALUE	FOLD_ENRICHMENT	# DE GENES
mmu01100	Metabolic pathways	2.06E-04	3.15	15
mmu00260	Glycine, serine and threonine metabolism	4.88E-03	19.58	3
mmu00270	Cysteine and methionine metabolism	6.22E-02	14.18	2
mmu04672	Intestinal immune network for IgA production	6.94E-02	11.54	2

Supplementary Table 7: Gene ontology (GO) biological process enrichment for 49 RORC target genes that were also DE in $H^{Cyp51-/-}$ runts.

ID_CATEGORY	NAME_CATEGORY	#DE GENES	FOLD_ENRICHMENT	ADJUSTED_PVALUE
GO:0009069	serine family amino acid metabolic process	3	33.47	3.42E-02
GO:0006565	L-serine catabolic process	2	215.73	3.42E-02
GO:0006082	organic acid metabolic process	9	5.17	3.42E-02
GO:0009063	cellular amino acid catabolic process	4	19.61	3.42E-02
GO:0046335	ethanolamine biosynthetic process	2	129.44	3.42E-02
GO:0042866	pyruvate biosynthetic process	2	129.44	3.42E-02
GO:0006646	phosphatidylethanolamine biosynthetic process	2	129.44	3.42E-02
GO:0006580	ethanolamine metabolic process	2	107.86	4.37E-02
GO:0043436	oxoacid metabolic process	8	4.72	4.64E-02
GO:0006563	L-serine metabolic process	2	80.9	4.64E-02
GO:1901162	primary amino compound biosynthetic process	2	80.9	4.64E-02
GO:0044281	small molecule metabolic process	13	3.01	4.64E-02
GO:0046337	phosphatidylethanolamine metabolic process	2	92.45	4.64E-02
GO:0019752	carboxylic acid metabolic process	8	4.72	4.64E-02
GO:0042180	cellular ketone metabolic process	8	4.56	5.09E-02
GO:0006520	cellular amino acid metabolic process	5	8.25	5.18E-02
GO:0051917	regulation of fibrinolysis	2	64.72	6.12E-02
GO:0046395	carboxylic acid catabolic process	4	10.7	6.86E-02
GO:0016054	organic acid catabolic process	4	10.7	6.86E-02
GO:0009070	serine family amino acid biosynthetic process	2	53.93	7.59E-02
GO:0009071	serine family amino acid catabolic process	2	46.23	9.93E-02

Supplementary Table 8: A list of used RT-qPCR primers and their respective sequences.

Gene name	Sequence	
	Sense	Antisense
Hmbs	TCCCTGAAGGATGTGCCTA	AAGGGTTTTCCCGTTTGC
Ppib	GGAGATGGCACAGGAGGAAA	CGTAGTGCTTCAGTTTGAAGTTCT
Cyp51	ACGCTGCCTGGCTATTGC	TTGATCTCTCGATGGGCTCTATC
Rora	TTACGTGTGAAGGCTGCAAG	GGAGTAGGTGGCATTGCTCT
Rorc	ACCTCTTTTCACGGGAGCA	TCCCACATCTCCCACATTG
Avpr1a	GGGATACCAATTTCGTTTGG	AAGCCAGTAACGCCGTGAT
Lipc	ACAAGGCGTGGGAACAGA	TGGCTTCTTTAATGGCTTGC

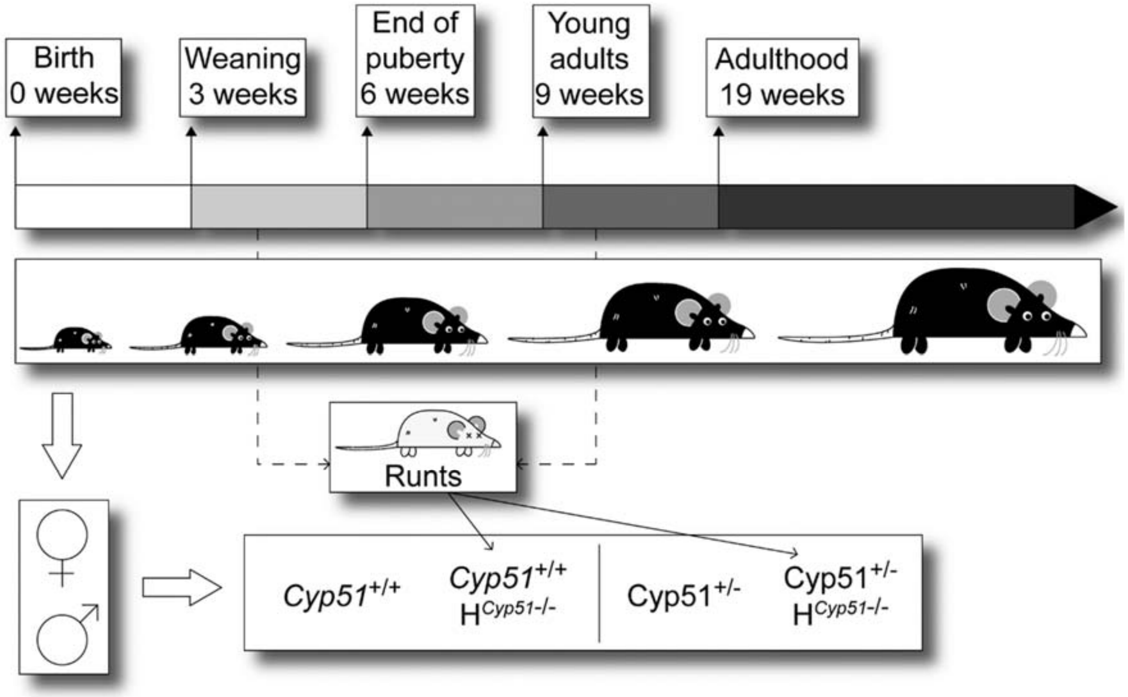

Supplementary Figure 1: Schematic representation of the experimental design. Mice of both sexes and the four genotypes were euthanized at the designated time points (0, 3, 6, 9 and 19 weeks). Runt mice were present only in the *Cyp51* knockout group, regardless of the *Cyp51* background. They appeared at 4–10 weeks of age and died or were euthanized at reasonably/humanely set time points.

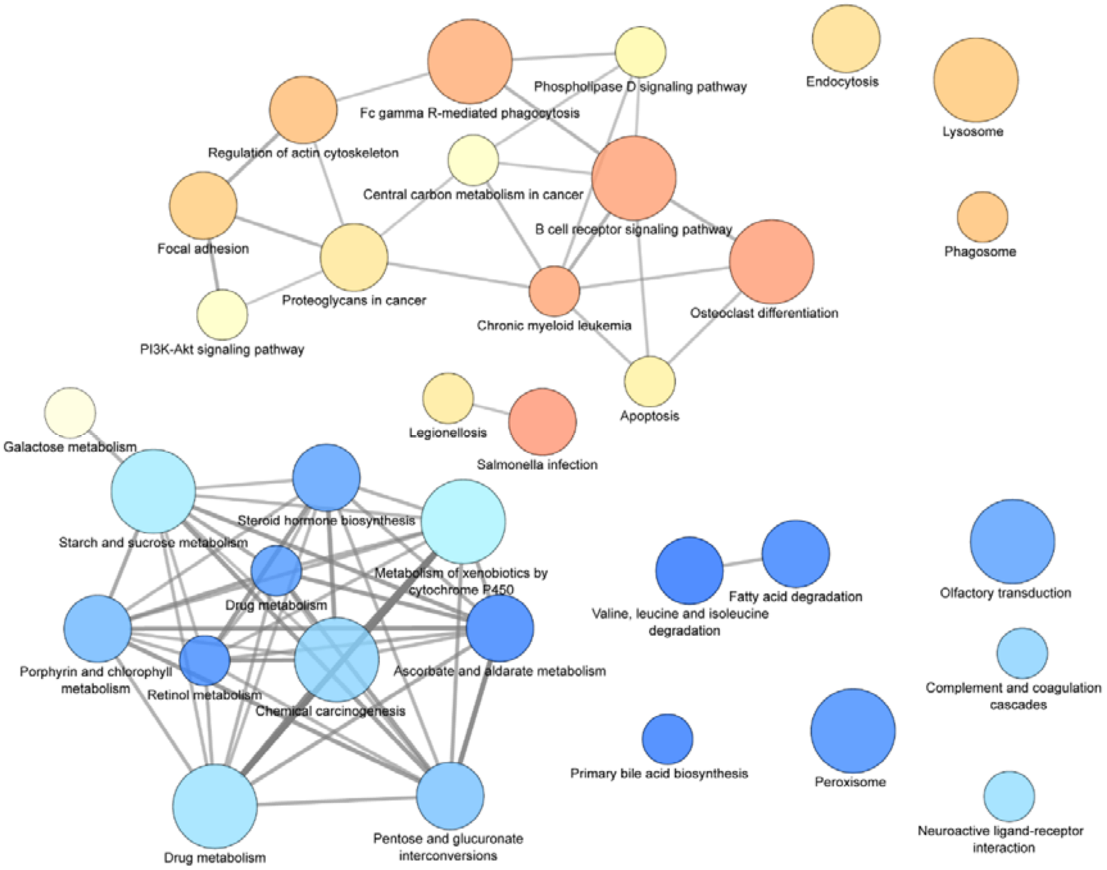

Supplementary Figure 2: Network of related KEGG pathways in H$^{Cyp51-/-}$ runts. KEGG pathway enrichment was done using Cytoscape and ClueGO plugin as described in the supplementary methods section. Orange colour depicts upregulated pathways, blue downregulated. Circle size correlates with the significance level ($p<0.05$ in all nodes). Colour intensity correlates to the overlap between the listed DE genes and the genes in a given pathway. Line width correlates with the degree of connectivity between the corresponding terms.

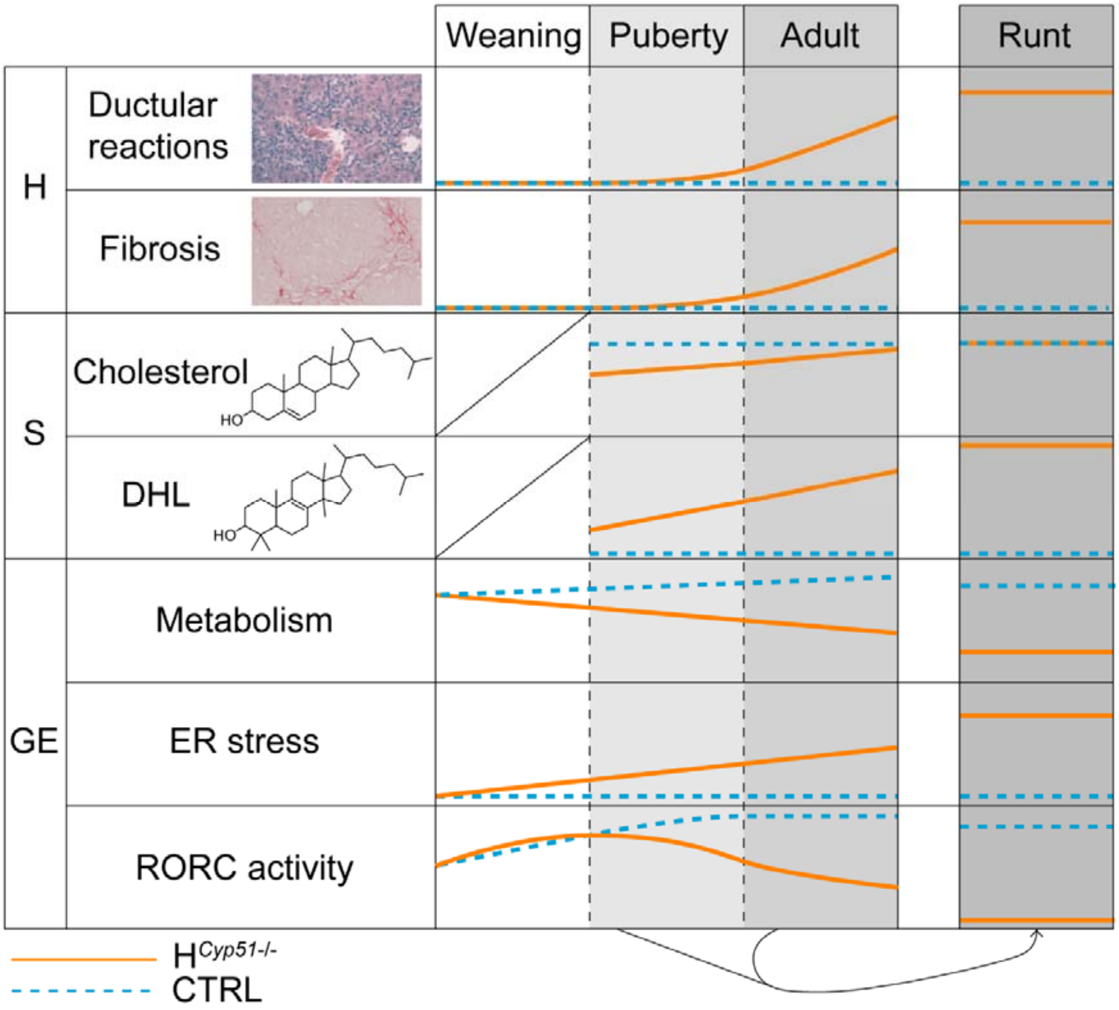

Supplementary Figure 3: Overview of the effects of hepatocyte *Cyp51* disruption on liver development. Schematic representation of the progressive changes observed during the development of the $H^{Cyp51-/-}$ mice (orange lines) in comparison with controls (blue dashed lines). H, histological features; S, liver sterol concentrations; GE, changes in gene expression.

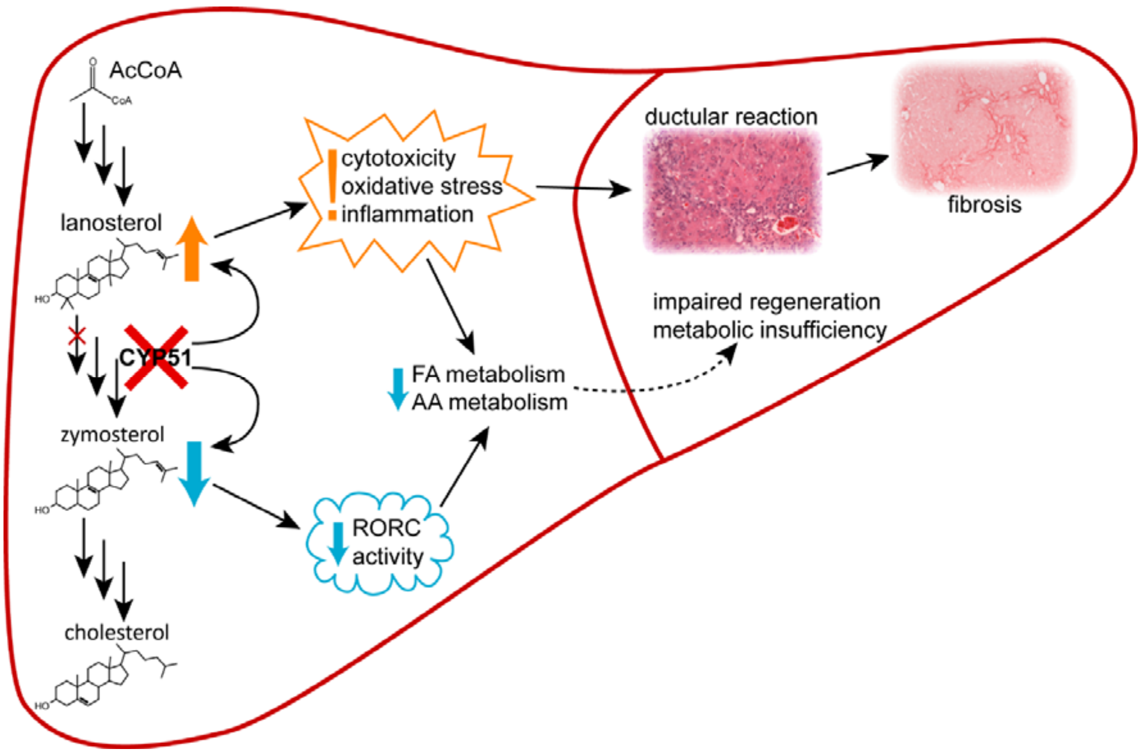

Supplementary Figure 4: The impact of *Cyp51* disruption on liver homeostasis. Shown is the proposed mechanism of disease development in H$^{Cyp51-/-}$ mice and the interaction between cholesterol synthesis and RORC pathways. AA – amino acid; FA – fatty acid.

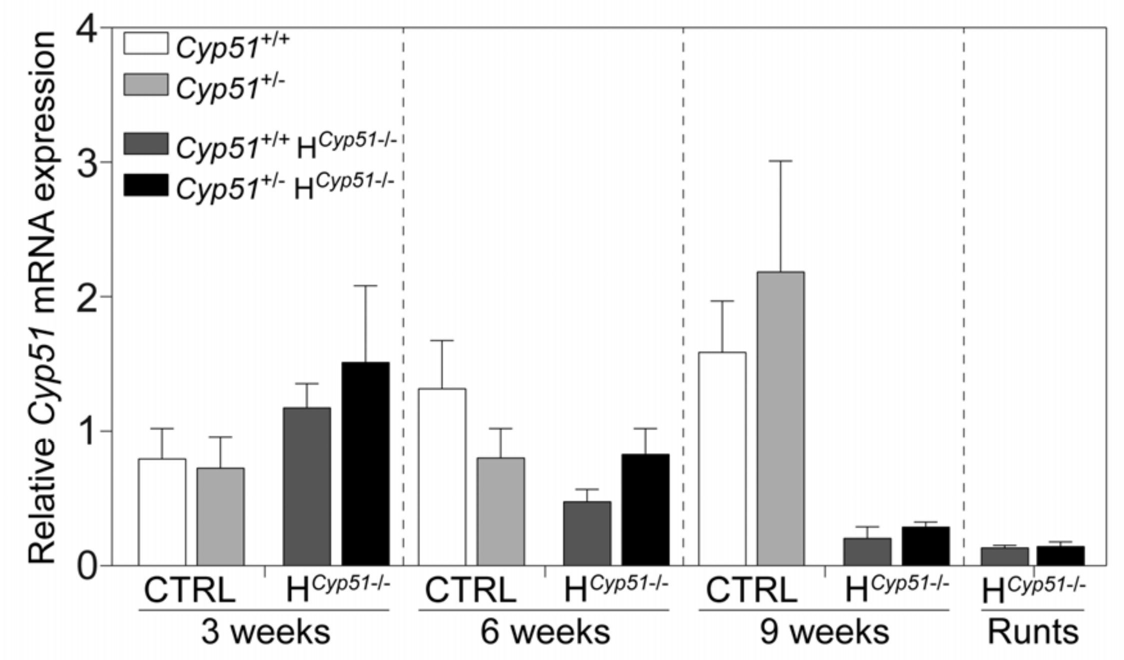

Supplementary Figure 5: The impact of *Cyp51* background on hepatic *Cyp51* gene expression. A comparison of the relative *Cyp51* expression between control, $H^{Cyp51-/-}$ and runt mice on the wild type ($Cyp51^{+/+}$) and heterozygous ($Cyp51^{+/-}$) background at different ages ($n \geq 4$). Columns depict means and error bars represent SEM.
